# Immunotherapy Using Immunogenic Mimotopes Selected by Phage Display plus Amphotericin B Inducing a Therapeutic Response in Mice Infected with *Leishmania amazonensis*

**DOI:** 10.3390/pathogens12020314

**Published:** 2023-02-14

**Authors:** Tauane G. Soyer, Fernanda F. Ramos, Isabela A. G. Pereira, Daniela P. Lage, Raquel S. Bandeira, Marcelo M. de Jesus, Guilherme P. Costa, Amanda S. Machado, Camila S. Freitas, Danniele L. Vale, Vívian T. Martins, Alexsandro S. Galdino, Miguel A. Chávez-Fumagalli, Daniel Menezes-Souza, Mariana C. Duarte, Bruno M. Roatt, Eduardo A. F. Coelho, Grasiele S. V. Tavares

**Affiliations:** 1 Programa de Pós-Graduação em Ciências da Saúde: Infectologia e Medicina Tropical, Faculdade de Medicina, Universidade Federal de Minas Gerais, Belo Horizonte 31270-901, Brazil; 2 Laboratório de Imunopatologia, Núcleo de Pesquisas em Ciências Biológicas/NUPEB, Departamento de Ciências Biológicas, Insituto de Ciências Exatas e Biológicas, Universidade Federal de Ouro Preto, Ouro Preto 35400-000, Brazil; 3 Laboratório de Biotecnologia de Microrganismos, Universidade Federal de São João Del-Rei, Divinópolis 35501-296, Brazil; 4 Computational Biology and Chemistry Research Group, Vicerrectorado de Investigación, Universidad Católica de Santa María, Urb. San José S/N, Umacollo, Arequipa 04000, Peru; 5 Departamento de Patologia Clínica, COLTEC, Universidade Federal de Minas Gerais, Av. Antônio Carlos, 6627, Belo Horizonte 31270-901, Brazil

**Keywords:** phage display, mimotopes, immunotherapeutics, tegumentary leishmaniasis, immune response, amphotericin B

## Abstract

*Leishmania amazonensis* can cause cutaneous and visceral clinical manifestations of leishmaniasis in infected hosts. Once the treatment against disease is toxic, presents high cost, and/or there is the emergence of parasite-resistant strains, alternative means through which to control the disease must be developed. In this context, immunotherapeutics combining known drugs with immunogens could be applied to control infections and allow hosts to recover from the disease. In this study, immunotherapeutics protocols associating mimotopes selected by phage display and amphotericin B (AmpB) were evaluated in *L. amazonensis*-infected mice. Immunogens, A4 and A8 phages, were administered alone or associated with AmpB. Other animals received saline, AmpB, a wild-type phage (WTP), or WTP/AmpB as controls. Evaluations performed one and thirty days after the application of immunotherapeutics showed that the A4/AmpB and A8/AmpB combinations induced the most polarized Th1-type immune responses, which reflected in significant reductions in the lesion’s average diameter and in the parasite load in the infected tissue and distinct organs of the animals. In addition, the combination also reduced the drug toxicity, as compared to values found using it alone. In this context, preliminary data presented here suggest the potential to associate A4 and A8 phages with AmpB to be applied in future studies for treatment against leishmaniasis.

## 1. Introduction

Leishmaniases are caused by protozoan parasites of the genus *Leishmania,* with more than 20 parasite species being able to cause visceral (VL) and tegumentary (TL) leishmaniasis [1]. VL can be fatal, if acute and left untreated. Otherwise, TL can cause from a self-limiting cutaneous lesion to disfiguring and destructive scars, causing patient morbidity [2]. In Brazil, TL is mainly caused by *Leishmania braziliensis*, *L. guyanensis*, and *L. amazonensis* species; however, *L. amazonensis* is particularly important, since this parasite species can cause both clinical manifestations of disease in infected hosts [3,4,5].

The treatment against leishmaniasis is based on the use of drugs, such as pentavalent antimonials, pentamidine, paramomycin, miltefosine, deoxycholate amphotericin B (AmpB) and its liposomal formulations, among others [6]. However, these compounds present limitations due to their toxicity, high cost, and/or emergence of resistant strains. In addition, after treatment, there is often the maintenance of residual parasites in tissues and organs of the hosts, suggesting that the infection reactivation can occur. Such facts are related to age, nutritional status, gender, comorbidities, coinfections, among others [7,8]. In this context, an alternative to subvert the immune profile related to the susceptibility to infection could be based on the association of treatment with the vaccine therapy, leading to the so-called immunotherapeutics procedures [9].

In fact, advances in the knowledge of immune response have led to a better understanding of disease pathogenesis, enabling the discovery of new forms of prophylaxis and therapeutics against leishmaniasis. In this case, the association between conventional drugs and preventive immunogens could revert to a more effective combination, restoring and/or inducing the development of a desired immune response in the infected mammalian hosts [10]. For instance, this strategic action has proven to be promising in canine models, where the reestablishing of animal immunity and parasite control have been achieved [11,12].

The Th1-type immune response directed against *Leishmania* parasite, reflected by the production of cytokines, such as IFN-γ, IL-2, TNF-α, IL-12, among others, is related to the resistance and protection against infection [13,14]. This immune response is related to the upregulation of antileishmanial activity of phagocytic cells, which is an important effector mechanism of parasite death [15]. In contrast, the establishment of Th2-type response, primed by presence in high levels of IL-4, IL-5, IL-6, IL-10, TGF-β, among others, is related to susceptibility in the development of active disease [16]. In this context, one could speculate that to achieve a successful immunotherapeutics strategy, the association between known drugs and immunogens able to induce a Th1-type response would be desired.

Recently, two phage-exposed mimotopes were selected by means of phage display technology, when sera and peripheral blood mononuclear cells (PBMCs) from TL patients were used [17]. They were called A4 and A8 and later evaluated as vaccine candidates in a murine model, with the purpose of verifying the protection induced against *L. amazonensis* infection. Results showed that A4- and A8-immunized animals developed a Th1-type response before and after infection, which reflected in significant reductions in the parasite load in the infected tissue and in distinct organs of the animals.

In the present study, A4 and A8 clones were evaluated in immunotherapeutics schedules against *L. amazonensis* infection. They were administered alone or associated with a reference drug, AmpB, and used to treat the infected animals. After the development of lesions, mice received the immunotherapeutic protocols, and immunological and parasitological analyses were performed at two distinct endpoints, one and thirty days after the treatments, aiming to verify a long-term action against infection. To the best of our knowledge, it is the first time that mimotopes identified by phage display are associated with an antileishmanial drug and used in immunotherapeutic protocols for the treatment of a mammalian host against TL.

## 2. Materials and Methods

### 2.1. Animals

The Committee on the Ethical Handling of Research Animals from Federal University of Minas Gerais (UFMG; Belo Horizonte, Minas Gerais, Brazil) approved the study with protocol number 144/2020. BALB/c mice (female, 6 to 8 weeks of age) were used in this work and they were maintained under specific pathogen-free conditions.

### 2.2. Parasites

*L. amazonensis* (IFLA/BR/1967/PH-8) promastigotes were cultured at 24 °C in complete Schneider’s medium (Sigma-Aldrich, St. Louis, MO, USA), which was composed by medium plus 20% heat-inactivated fetal bovine serum (FBS; Sigma-Aldrich, USA), 20 mM L-glutamine, 200 U/mL penicillin, and 100 µg/mL streptomycin, at pH 7.4. The soluble *Leishmania* antigenic extract (SLA) was prepared, as described elsewhere [18].

### 2.3. Amplification of the Phage Clones

To obtain the A4 and A8 phages, clones were amplified and purified according to previously described findings [17]. A wild-type phage (WTP), which did not express a foreign peptide, was also amplified and used as molecule control. The concentration of the purified phages was estimated in a spectrophotometer reader at 600 nm and, after amplification, they were stored in a 50% glycerol solution at −20 °C, until use.

### 2.4. Preparation of the Parasites and Infection

The animals’ infection was performed using stationary-phase promastigotes, which were cultured as described above. The parasite infectivity was maintained by serial passages in mice in our laboratory, and they were obtained after few passages (until the 4th in vitro passage), subsequently purified by filtering and inoculated (1 × 10^6^ promastigotes per animal) subcutaneously in the base of the tail of healthy mice. In addition, before infection, stationary promastigotes were previously viewed under optical light microscopy, and the cell density was estimated by being counted in a Newbauer chamber, with their morphology being evaluated after staining by Giemsa.

### 2.5. Immunotherapeutics Schedules

After the development of ulcerated lesions, which occurred at approximately 50 to 60 days post-infection, mice (*n* = 16 per group) were joined according to lesion size (2 to 3 mm) and received three interventions by subcutaneous route near the site of infection, with a seven day-interval between them, containing one of the following regimens: (i) saline group: mice received 50 μL of PBS; (ii) WTP group: mice received only the WTP clone (1 × 10^10^ phages) diluted in PBS; (iii) AmpB group: mice received only AmpB (1 mg/kg/weight); (iv) WTP/AmpB group: mice received WTP (10^10^ phages) plus AmpB (1 mg/kg/weight); (v) A4 group: mice received only the A4 clone (10^10^ phages) diluted in PBS; (vi) A4/AmpB group: mice received A4 (10^10^ phages) plus AmpB (1 mg/kg/weight); (vii) A8 group: mice received only the A8 clone (10^10^ phages) diluted in PBS; and (viii) A8/AmpB group: mice received A8 (10^10^ phages) plus AmpB (1 mg/kg/weight). During and after the administration of the immunotherapeutics; the footpad swelling was measured weekly using an electronic caliper (799-6/150 model, Starrett^®^, Itu, Brazil). Animals were euthanized one (*n* = 8 per group) and thirty (*n* = 8 per group) days after treatments, and their infected tissue, spleen, liver, draining lymph nodes (dLNs), and blood samples were collected.

### 2.6. Evaluation of Cell Response

#### 2.6.1. Capture ELISA and Nitrite Production

Spleen cells (5 × 10^6^ per well) were collected from euthanized animals in both periods of time (*n* = 8 per group, in each step), and were plated in complete RPMI 1640 medium in duplicate in 24-well plates (Nunc). Cells were stimulated with each phage used in the mice group (10^10^ phages) or with their mixture in the saline and AmpB groups. Cells were also stimulated with SLA (25 μg/mL) for 48 h at 37 °C in 5% CO_2_. IFN-γ, IL-4, IL-10, and IL-12 levels were measured in the culture supernatant by a capture ELISA (catalogs 555138, 555232, 555252, and 555256, respectively; OptEIA TM set mouse kits; all acquired from BD Pharmingen, San Diego, CA, USA). The culture supernatant was also used to evaluate the nitrite presence by Griess reaction, when 50 µL of supernatant were mixed with an equal volume of Griess reagent, and the nitrite concentration was calculated from a standard curve, with absorbances being measured at 540 nm.

#### 2.6.2. Polyfunctional T-cell Analysis by Flow Cytometry

Spleen cell cultures were also used to evaluate cell surface markers and intracellular cytokine (IFN-γ, TNF-α, IL-2, and IL-10), as previously described [19]. Antibodies against IL-2 (PE anti-mouse, clone JES6-5H4, catalog 554428), IFN-γ (AF700 anti-mouse, clone XMG1.2, catalog 557998), TNF-α (PE-Cy7 anti-mouse, clone LG.3A10, catalog 557644), and IL-10 (APC anti-mouse, clone JES5-16E, catalog 554468) were used. Cells were acquired (100,000 events) on an LSR Fortessa cytometer (BD Biosciences, USA) using FACSDiva software, and data were expressed as indexes, which were calculated by the ratio between the percentages of positive cells found in the stimulated cultures versus those found in the unstimulated cultures.

#### 2.6.3. RNA Extraction and Real-Time-qPCR (RT-qPCR)

The IFN-γ expression was evaluated in the stimulated cell cultures (*n* = 8 mice per group), 30 days after the immunotherapeutics, as previously described [20]. Results were shown graphically as fold changes in gene expression by using the mean ± standard deviation of the target gene. Data were analysed according to the relative expression using the 2^–ΔΔCT^ method.

### 2.7. Humoral Response

Anti-phage and anti-SLA IgG1 and IgG2a antibodies were evaluated in the treated animals in both periods of time (*n* = 8 mice per group, in each step), as previously described [21]. Briefly, microtiter plates (Jetbiofil^®^, Belo Horizonte, Minas Gerais, Brazil) were coated with A4, A8, WTP, or SLA (10^10^, 10^10^, 10^10^, and 1.0 µg per well, respectively) in 50 mM carbonate buffer at pH 9.6, for 18 h at 4 °C. Free binding sites were blocked using PBS-T [PBS and Tween 20 0.05% plus 5% (*w*/*v*) bovine serum albumin) for 1 h at 37 °C. Plates were washed in PBS-T and incubated with individual sera (1:100 diluted in PBS-T) for 1 h at 37 °C. They were again washed in PBS-T and incubated with anti-mouse IgG1 or IgG2a peroxidase-conjugated antibodies (catalogs SA1-35640 and SA1-35646, respectively; Invitrogen, Waltham, MA, USA). Titration curves for IgG1 and IgG2a peroxidase-conjugated antibodies were done using pooled sera and dilutions varying from 1:1000 to 1:80,000, which were performed in PBS-T. A new incubation was performed for 1 h at 37 °C, when plates were washed with PBS-T and reactions were developed by adding a solution comprised of H_2_O_2_, ortho-phenylenediamine and citrate-phosphate buffer at pH 5.0 for 30 min and in the dark. Reactions were stopped by adding 2N H_2_SO_4_, and optical density (OD) values were read in spectrophotometer at 492 nm.

### 2.8. In Vivo Toxicity

Toxicity was evaluated at one and thirty days after the immunotherapeutics procedures. For this, sera were collected (*n* = 8 mice per group, in each step) and levels of alanine transaminase (ALT) and aspartate transaminase (AST) enzymes, as hepatic damage markers, and urea and creatinine, as renal damage markers, were measured using commercial kits (Labtest Diagnostica^®^, Belo Horizonte, Minas Gerais, Brazil), following manufacturer instructions. Samples from uninfected and untreated mice (*n* = 5) were used as control.

### 2.9. Evaluation of Parasite Burden

Organic parasitism was evaluated in the infected tissue, liver, spleen, and dLNs from treated animals, in both periods of time (*n* = 8 mice per group, in each step), by a limiting dilution technique and according to that described elsewhere [21]. Results were expressed as the titer negative log adjusted per milligram of tissue and organ. In addition, splenic parasitism was also evaluated in the animals (*n* = 8 mice per group), 30 days after the immunotherapeutics, by applying a qPCR technique [21]. Results were calculated by interpolation from a standard curve included in the same run, which was performed in duplicate and expressed as the number of parasites per total DNA.

### 2.10. Statistical Analysis

Microsoft Excel (version 10.0) spreadsheets and GraphPad Prism^TM^ were used to evaluate the results. The one-way analysis of variance (ANOVA) and the paired Student’s *t* test were used to evaluate results between the groups. Differences were considered significant with *p* < 0.05.

## 3. Results

### 3.1. Cell Response Generated after the Immunotherapeutics Procedures

**Figure 1:**
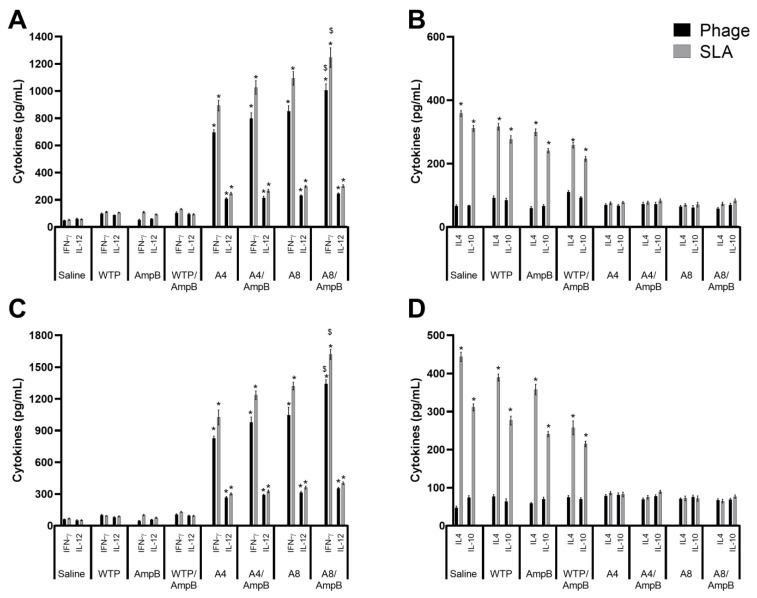
Cytokine response evaluated one and thirty days after immunotherapeutics. Mice (*n* = 16 per group) were infected and later received saline, amphotericin B (AmpB), wild-type phage (WTP), WTP/AmpB, A4, A4/AmpB, A8, or A8/AmpB. One and thirty days after immunotherapeutics, animals´ spleen cells (*n* = 8 mice per group, in each step) were cultured (5 × 10^6^ cells per well) in complete RPMI 1640 medium, at which time they were stimulated with the A4, A8, wild-type (WTP) (10^10^ phages, each), or SLA (25 µg/mL) for 48 h at 37 °C in 5% CO_2_. IFN-γ, IL-12, IL-4, and IL-10 levels were then measured in culture supernatant by a capture ELISA. Results obtained one (**A**,**B**) and thirty (**C**,**D**) days after the immunotherapeutics are shown as bars, which indicate the mean ± standard deviation of the groups. * indicates significant difference in relation to the saline, AmpB, WTP, and WTP/AmpB groups (*p* < 0.0001). ^$^ indicates significant difference in relation to the A4, A4/AmpB, and A8 groups (*p* < 0.01).

The cytokine response was evaluated in *L. amazonensis*-infected mice. Results obtained one day after the immunotherapeutics suggested that mice receiving the A4/AmpB and A8/AmpB combinations developed a polarized Th1-type cell response, with higher levels of IFN-γ and IL-12 found in the culture supernatants, which were associated with low production of IL-4 and IL-10, after stimuli using the phage or SLA (Figure 1A). Thirty days after the procedures, the Th1-type cell response profile was also found in these animals (Figure 1B). The combination between phage and AmpB resulted in higher IFN-γ and IL-12 production, as compared to the use of phage alone. On the other hand, control mice developed a Th2-type response in both periods of time (Figure 1).

**Figure 2:**
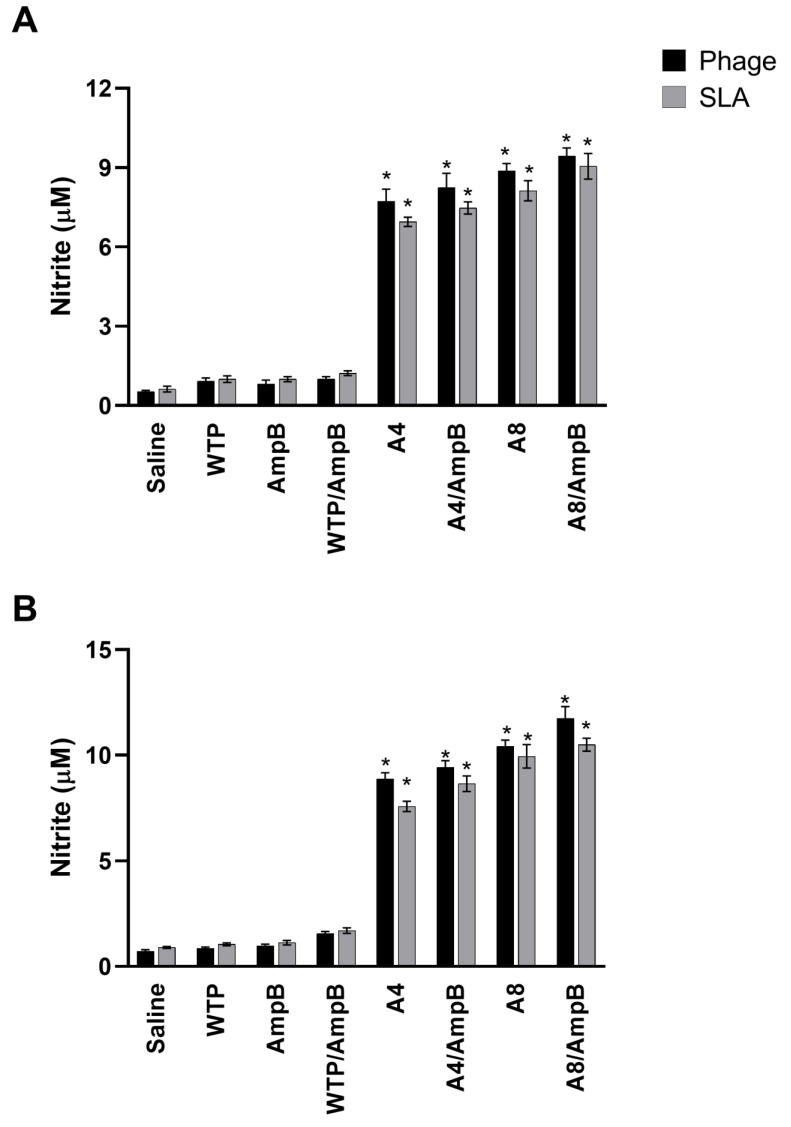
Nitrite secretion evaluated one and 30 days post-schedules. The cell culture supernatant used to evaluate the cytokine production was also used to evaluate the nitrite presence by Griess reaction, one (**A**) and thirty (**B**) days after the procedures. Bars indicate the mean ± standard deviation of the groups. * indicates significant difference in relation to the saline, amphotericin B (AmpB), wild-type phage (WTP), and WTP/AmpB groups (*p* < 0.0001).

The nitrite presence was evaluated as a cell activation marker, and results showed that the A4/AmpB and A8/AmpB combinations induced a higher production of this antileishmanial molecule, when compared to the others. Although the nitrite production also proved to be high in mice receiving the phages alone, it was lower as compared to the use of the association between the clones and AmpB, in both evaluated periods of time (Figure 2).

**Figure 3:**
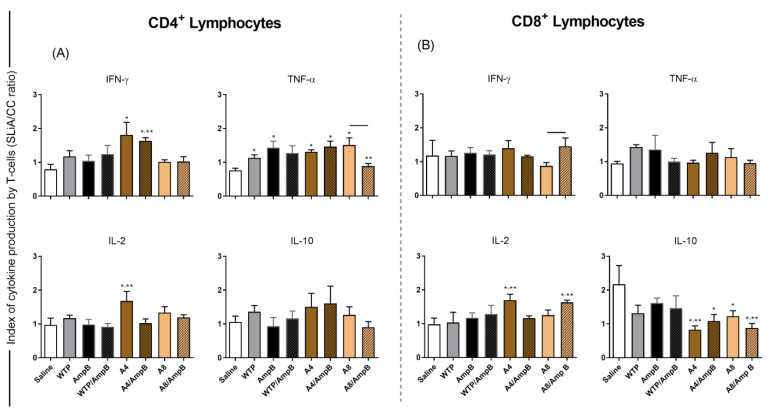
Intracytoplasmic cytokine-producing CD4^+^ and CD8^+^ T-cell frequency. Spleen cells were obtained from mice (*n* = 8 per group) thirty days after the immunotherapeutics, and cells (5 × 10^6^ per well) were stimulated with SLA (25 µg/mL) for 48 h at 37 °C in 5% CO_2_. The x-axis shows the groups, while the y-axis indicates the index values, which were expressed by the ratio between the IFN-γ, TNF-α, IL-2, and IL-10-producing a CD4^+^ (**A**) and CD8^+^ (**B**) T-cell frequency in the stimulated cultures as compared to unstimulated cultures (SLA/CC ratio). Bars indicate the mean plus standard deviation of the groups. * and ** indicate significant difference (*p* < 0.05) in relation to the saline and amphotericin B (AmpB) groups, respectively, and by connecting lines.

In addition, a flow cytometry assay showed that A4/AmpB and A8/AmpB groups presented higher CD4^+^ T-cell frequency producing IFN-γ and IL-2, when compared to the control groups. A similar profile was found when CD8^+^ T cells were evaluated. Otherwise, the IL-10-producing T-cell frequency proved to be higher in the saline, AmpB, WTP, and WTP/AmpB groups mice, when compared to those receiving A4/AmpB or A8/AmpB (Figure 3).

**Figure 4:**
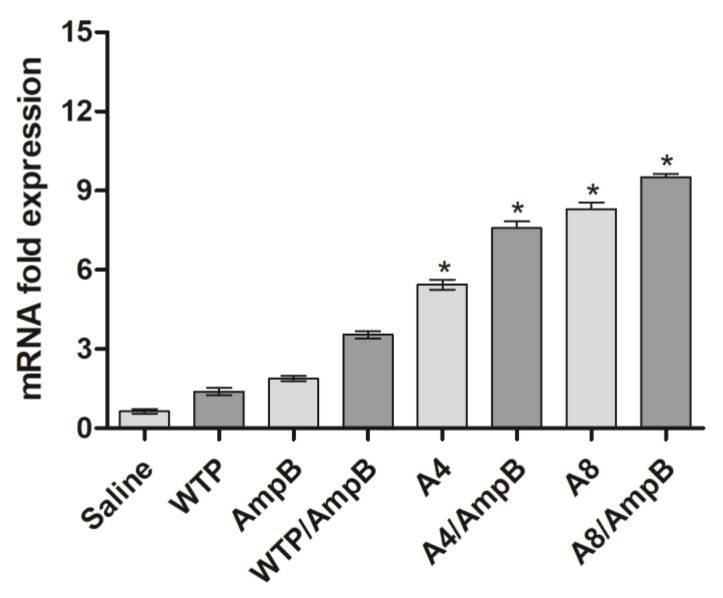
IFN-γ mRNA expression. Spleen cells were obtained from mice (*n* = 8 per group) 30 days after the immunotherapeutics, and cells (5 × 10^6^ per well) were stimulated with SLA (25 µg/mL) for 48 h at 37 °C in 5% CO_2_. The RNA content was then extracted in order to evaluate the IFN-γ expression in the stimulated cultures by a RT-qPCR technique. Bars indicate the mean ± standard deviation of groups. * indicates a significant difference in relation to the saline, amphotericin B (AmpB), wild-type phage (WTP), and WTP/AmpB groups (*p* < 0.0001).

The IFN-γ expression was also evaluated in the stimulated cell cultures, and results showed that stimulated cell cultures from A4/AmpB and A8/AmpB groups presented higher levels of anti-SLA IFN-γ mRNA, as compared to data found in the controls (Figure 4). In addition, mice receiving A4 and A8 alone also showed higher IFN-γ expression, when compared to values found in the saline, WTP, AmpB, and WTP/AmpB groups; although they were lower in comparison to data found in the A4/AmpB and A8/AmpB groups.

### 3.2. Humoral Response Developed after the Immunotherapeutics

**Figure 5:**
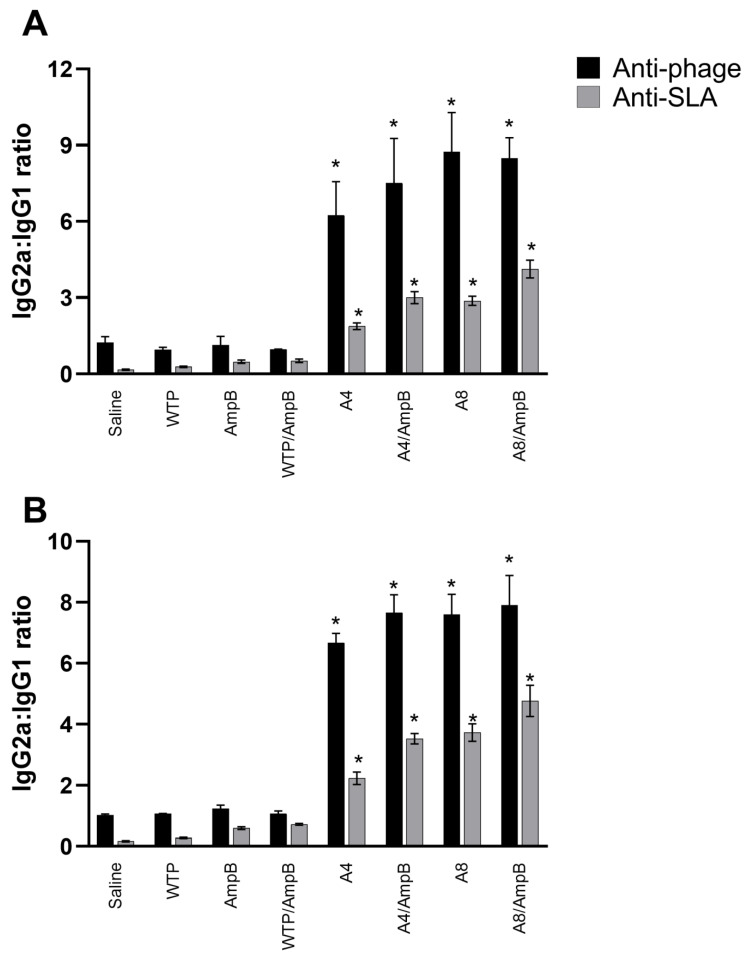
Ratios of IgG1 and IgG2a isotype levels found one and thirty days after the immunotherapeutics. Mice (*n* = 16 per group) were infected and later received saline, amphotericin B (AmpB), wild-type phage (WTP), WTP/AmpB, A4, A4/AmpB, A8, or A8/AmpB. One and thirty days (*n* = 8 per group in each step) after immunotherapeutics, their sera samples were collected, and the presence of anti-phage and anti-SLA IgG1 and IgG2a isotype antibodies was evaluated. With the optical density values, ratios between IgG2a and IgG1 isotypes were calculated, and results are shown one (**A**) and thirty (**B**) days after the procedures. Bars indicate the mean ± standard deviation of the groups. * indicates a significant difference in relation to the saline, AmpB, WTP, and WTP/AmpB groups (*p* < 0.0001).

The anti-phage and anti-SLA humoral response was evaluated, and results showed that when sera were collected from animals one day after the immunotherapeutics, A4/AmpB and A8/AmpB groups presented higher IgG2a and lower IgG1 levels, which were reflected in higher ratios between these antibody isotypes (Figure 5), indicating the development of a Th1-type humoral response in these animals. Otherwise, control mice presented lower IgG2a and higher IgG1 levels, which were reflected in lower ratios between IgG2a and IgG1 isotypes and that correlated with the occurrence of a Th2-type response. Titration curves were performed for IgG isotype conjugates, using samples collected one and thirty days after the immunotherapeutics, and results showed in both periods of time (Table 1 and Table 2, respectively) reflected the maintenance of the reactivity reduction according to the antibody dilution, as well as the reaction linearity and proportion between the IgG isotypes.

### 3.3. In Vivo Toxicity Evaluated in the Treated and Infected Mice

Renal and hepatic damage markers were evaluated in animals’ sera, and results showed that, in both periods of time, lower levels of AST, ALT, urea, and creatinine were found in the A4/AmpB and A8/AmpB groups, when compared to values found in the others (Table 3). On the other hand, saline and AmpB groups mice presented higher levels of these organic damage markers, when compared to the others, reflecting both the infection effect and drug toxicity.

### 3.4. Evolution of L. amazonensis Infection in the Treated Mice

**Figure 6:**
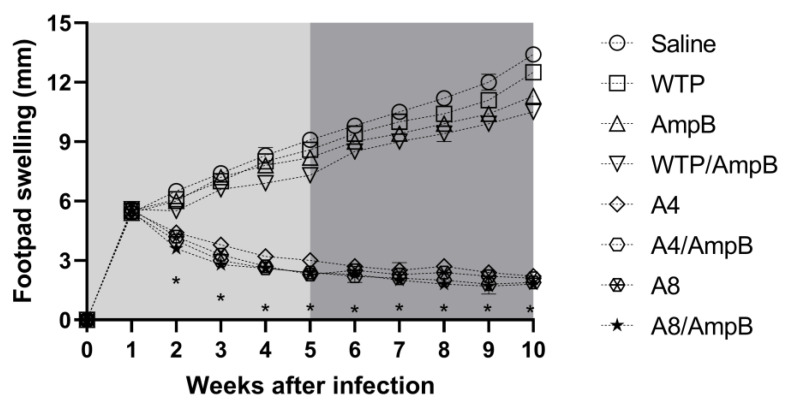
Lesion development during and after the immunotherapeutics. Mice were infected with *L. amazonensis* promastigotes and later received saline, amphotericin B (AmpB), wild-type phage (WTP), WTP/AmpB, A4, A4/AmpB, A8, or A8/AmpB. During and after the immunotherapeutics, the footpad swelling was measured weekly, and expressed as the increase in average lesion diameter from 16 (0 to 5th week) and 8 (6th to 10th) mice per group, when the immunotherapeutics were begun. Lines indicate the mean of the groups. * indicates significant difference in relation to the saline, amphotericin B (AmpB), wild-type phage (WTP), and WTP/AmpB groups (*p* < 0.001).

The immunotherapeutic effect in the mice groups was evaluated by means of the measurement of the lesion’s average diameter and the parasite load in the animals. Results showed that A4/AmpB and A8/AmpB-receiving mice groups presented more significant reductions in the lesion average diameter, as compared to data obtained in the controls (Figure 6). Animals receiving the phages alone also presented a reduction in the lesion’s diameter, when compared to results obtained in the saline, AmpB, WTP, and WTP/AmpB groups; however, values were higher when compared to those obtained in the A4/AmpB and A8/AmpB groups.

**Figure 7:**
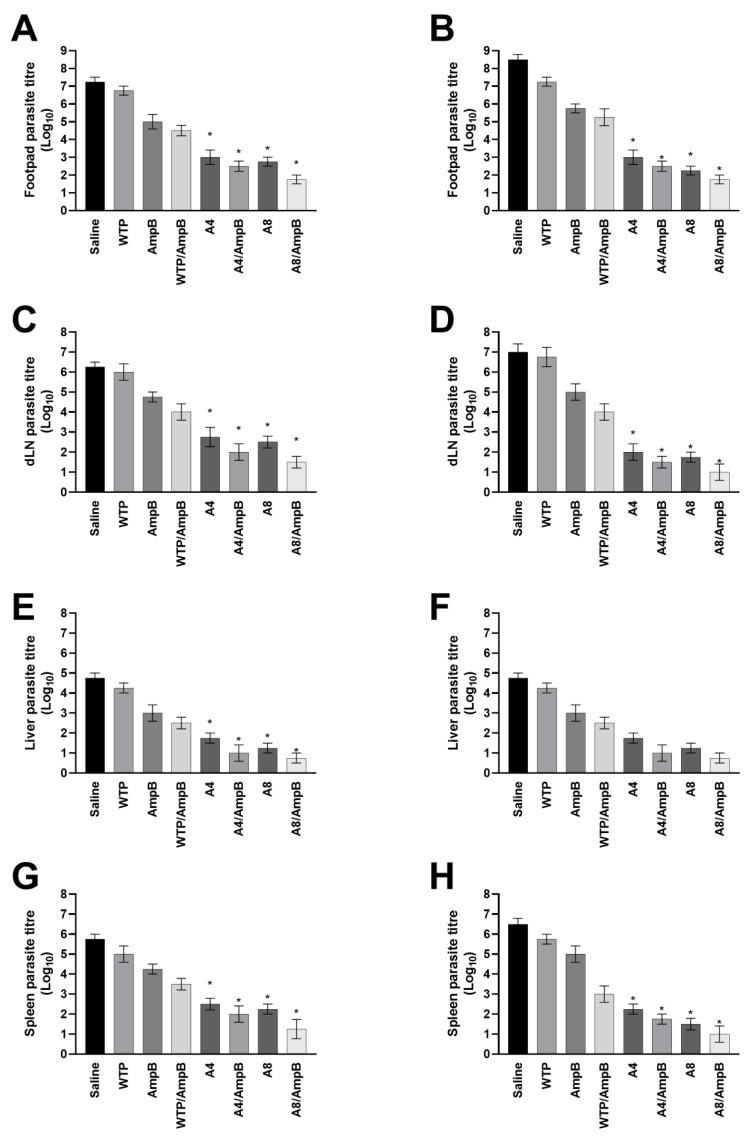
Parasite burden after the immunotherapeutics. Mice (*n* = 16 per group) were infected and later received saline, amphotericin B (AmpB), wild-type phage (WTP), WTP/AmpB, A4, A4/AmpB, A8, or A8/AmpB. One (*n* = 8 per group) and thirty (*n* = 8 per group) days after the immunotherapeutics, infected tissues, livers, spleens, and draining lymph nodes (dLNs) were collected of the animals, in order to evaluate the parasite load by a limiting dilution technique. Results obtained one (**A**,**C**,**E**,**G**) and thirty (**B**,**D**,**F**,**H**) days after the immunotherapeutics are shown as bars, which represent the mean ± standard deviation of the groups. * indicates significant difference in relation to the saline, AmpB, WTP, and WTP/AmpB groups (*p* < 0.0001).

**Figure 8:**
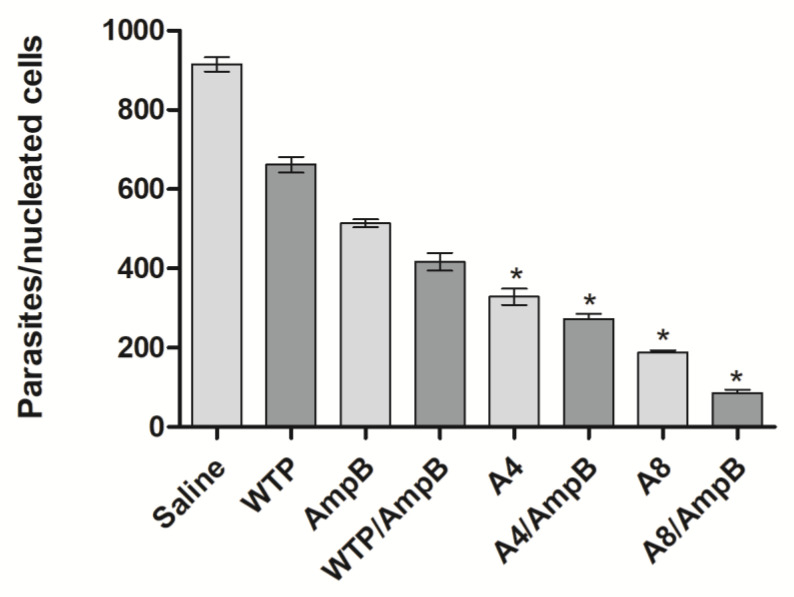
Splenic parasitism evaluated by qPCR. The parasite load was also evaluated in the animals’ spleens (*n* = 8 mice per group), 30 days after immunotherapeutics. Bars indicate the mean ± standard deviation of the groups. * indicates significant difference in relation to the saline, amphotericin B (AmpB), wild-type phage (WTP), and WTP/AmpB groups (*p* < 0.0001).

Similarly, the evaluation of parasite load showed that the A4/AmpB and A8/AmpB combinations induced more significant reductions in the animals’ parasitism, when compared to data found in the other groups, in both periods of time (Figure 7). The phages alone also induced significant reductions when compared to values found in the saline, AmpB, WTP, and WTP/AmpB groups. As an additional parameter to evaluate the parasite load, a qPCR technique was employed using animal splenic samples, and results showed that A4/AmpB and A8/AmpB groups also presented more significant reductions in the parasite load, when compared to the other groups, when analyses were performed 30 days after the procedures (Figure 8).

## 4. Discussion

The toxicity of antileishmanial drugs is a limiting factor to the success of the therapeutics. Furthermore, the occurrence of resistant strains and the high cost of AmpB-based liposomal formulations have also created limits to successful therapies against the disease [22]. Therefore, the search for new prophylactic and/or therapeutic strategies against this disease is urgent. Immunotherapeutics have gained importance in this aspect, since this tool associates the elimination of parasites by conventional drugs and the activation from host immune response by use of immunogens [23]. In this context, such association could contribute to the development of a synergistic effect, which could reflect a higher efficiency in eliminating parasites in a more effective manner and with a lower cost and time [24].

In the present study, we performed immunotherapeutics procedures based on the combination between immunogenic mimotopes, which were previously selected by phage display, and a reference antileishmanial drug, AmpB, seeking to evaluate the efficacy of this combination against the infection caused by *L. amazonensis* in a susceptible murine model. Preliminary results obtained here suggested that the A4/AmpB and A8/AmpB combinations induced a more polarized Th1-type immune response in the animals, which indicated a lower parasite load in the infected tissue and organs evaluated in this study. We have used drug and phage concentrations that were previously employed in treatment and vaccine studies developed by our group [25,26]. However, an interesting aspect will be the definition, which could be based on performing dose-response curves, from a minimal amount of AmpB capable of reducing parasite loads on its own, without causing significant organic toxicity to the hosts. It could then be combined with mimotopes in order to achieve the maximum effectiveness of the immunotherapeutics. In this context, data presented here are preliminary and additional experiments should be performed to solve such questions.

Phage-displayed mimotopes present advantages to use as biologicals in mammalians, since they are not pathogenic or toxic, and can even replicate inside host cells and potentiate the immune response. In addition, phage molecules are easier and cheaper to produce, when compared to the production of recombinant proteins. The final product also presents a high yield of purification [27]. In fact, a host’s protection against *Leishmania* infection commonly requires the induction of both CD4^+^ and CD8^+^ T cell subtypes, which produce pro-inflammatory cytokines and aid in the elimination of the parasites [28]. In this aspect, mimotopes also present advantages, when applied as vaccine candidates, since the molecules are taken up by host cells and presented in association with MHC classes I and II molecules for both T-cell subtypes [29]. In fact, CD4^+^ and CD8^+^ T cells are considered to be potent effector immune cells, which produce cytokines and aid the host’s immune system to kill parasites [30]. In our study, a flow cytometry assay showed that the A4/AmpB and A8/AmpB combinations induced a higher presence of both T-cell subtypes, in turn producing IFN-γ and IL-2. This fact is relevant and corroborates with the efficacy of mimotopes in inducing immune response by both T cells. Additionally, phage molecules contain cytosine-phosphate-guanosine motifs, which are recognized by Toll-like receptors present in a high number of antigen-presenting cells and which contribute to the production of pro-inflammatory cytokines [31]. All of these facts suggest that the association of immune adjuvants is not required, thus contributing to reduce production costs and product toxicity, such as that caused by saponins in mammalian hosts [32,33].

AmpB is a known antileishmanial drug, even its low specificity causes toxicity to mammalian cells [34]. AmpB-containing liposomal formulations have obtained better results in terms of safety and toxicity, even though these products entail high costs, which tends to hinder their use in endemic countries, where leishmaniasis is a neglected tropical disease [35]. In our study, we also observed that mice receiving AmpB alone developed renal and hepatic toxicity, despite having reduced the organic parasitism when compared to other controls. However, a surprising and synergic effect was found when AmpB was associated with the A4 or A8 phages, since treated animals who have received these phages presented a significant decline in the parasite load, which was associated with lower in vivo toxicity. This fact may well be related to a protective action from phage capsids to the host’s organic system [36]. Moreover, in spite of the direct action of AmpB in *Leishmania* parasites, the synergic development and activation of immune cells to differentiate in Th1-type cell populations, as well as to produce cytokines that contributed to a concomitant reduction in parasitism, could also reflect the lower toxicity found in these animals.

In our study, the use of A4 and A8 phages alone also led to IFN-γ and IL-12 production in the stimulated spleen cell cultures, thus suggesting an activation of the host’s immune system associated with a reduction in parasitism. These results were also associated with a higher nitrite secretion found in the cell supernatants. However, when the phages were used in association with AmpB, the cell response was more polarized, with higher IFN-γ and IL-12 production being found in the culture supernatants. This fact reflected in a lower parasite load in distinct organs, suggesting a better immunotherapeutics performance to the infected animals [37]. In other studies, the association between antileishmanial drugs, which also directly reduced parasitism in infected hosts, and immunogens, which subsequently contributed to the development of a protective immune response, also played a more effective therapeutic role when compared to the use of products administered separately [38,39,40].

BALB/c mice are a widely used experimental model to evaluate vaccine and immunotherapeutics candidates against leishmaniasis. In fact, these animals have proven to be useful to evaluate parasitism in distinct organs, indicating that evaluations in this mammalian model could be relevant to evaluate the parasitological and immunological responses caused by infection with *Leishmania* parasites [41]. This mouse lineage presents susceptibility to distinct parasite species, enabling the dissemination of *Leishmania* in organs such as the liver, which will later reduce at later stages of infection [42]. With such evolution, the hepatic parasitism tends to reduce with subsequent immunity to re-infection, whereas the splenic parasite load tends to increase and persists, when in the chronicity of infection [43]. However, despite its benefits, there are technical and scientific limitations to the applicability of this mouse model to other mammalian models, such as hamsters, dogs and humans and, in this context, they should also be evaluated before a product can be properly described as an effective vaccine and/or immunotherapeutics candidate [44,45]. Consequently, data presented here are preliminary and suggest that our combination involving mimotopes and AmpB showed high efficacy in a murine model. Nevertheless, new studies in other models should also be performed to confirm these biological actions.

Treatment of leishmaniasis presents distinct problems, and a successful vaccine has not yet been developed to protect mammalian hosts. The disease is mainly endemic in poor and developing countries. In this context, big pharmaceutical companies tend to neglect this type of treatment. As a consequence, innovative solutions should be formulated in an attempt to diminish the disease’s spectrum in these specific locations and their populations, and the association from known, although toxic, antileishmanial drugs, together with immunogens, could provide an effective solution [34]. In fact, drugs able to directly reduce parasitism in treated hosts, if associated with Th1-type immune inductors, could alter the disease phenotype in many patients. In one study, the association between allopurinol and a defined subunit vaccine in *L. infantum*-infected dogs showed that animals presented improved clinical conditions and provided a long-term clearance of parasites, when compared to the use of the drug or vaccine alone [46]. In our study, the A4 or A8 phages plus AmpB also presented better parasitological and immunological results when compared to the use of these products alone. The drug toxicity was also reduced by the association, possibly suggesting a protective organic role induced by the phage molecules. In this context, although other studies evaluating variations in such immunotherapeutics combinations should be performed, this experimental strategy can be considered a promising therapeutic and preventive path in a future.

The present study evaluated the efficacy of the products at two endpoints, one and thirty days after the immunotherapeutics procedures. Results obtained when immunological, biochemical, and parasitological analyses were performed in both periods showed that values appeared to be similar between the prior and later analyses, although a more polarized Th1-type response and lower parasitism have been found with experiments performed 30 days after immunotherapeutics therapy. This fact suggests an improvement, even if discrete, of the cell response profile and protection obtained against infection. However, additional assays should be also performed between one and thirty days, or more, in the treated animals, aiming to define the long-term protection and to evaluate a possible sterile cure. This work also presents specific limitations, and results presented here should be considered as a proof-of-concept of the effect of the association between immunogenic mimotopes and a well-known antileishmanial drug as a new immunotherapeutics procedure, which should eventually be evaluated in more refined combinations, seeking to diminish the number of doses and their concentrations, and produce new tests of this procedure in other mammalian hosts.

## Figures and Tables

**Table 1:** Titration curves performed for anti-SLA IgG1 and IgG2a peroxidase-conjugated antibodies using serum samples collected one day after the immunotherapeutics. Sera samples were collected from animals (*n* = 8 per group), one day after the immunotherapeutics procedures, and used in ELISA to evaluate the anti-parasite humoral reactivity. Microtiter plates were coated with *L. amazonensis* SLA (1.0 µg per well), reacted against pooled sera (1:100 diluted in PBS-T) and against IgG1 or IgG2a peroxidase-conjugated antibodies, which were 1:1000 to 1:80,000 diluted in PBS-T. Colorimetrical reactions were developed, stopped and read in a spectrophotometer at 492 nm. The mean of optical density (OD) value for each antibody dilution and mouse group is shown.

	Saline Group						
Isotype	1:1000	1:2500	1:5000	1:10,000	1:20,000	1:40,000	1:80,000
IgG1	2.133	1.545	0.877	0.418	0.217	0.112	0.065
IgG2a	0.443	0.244	0.133	0.069	0.035	0.017	0.010
	Wild-type phage (WTP) group						
Isotype	1:1000	1:2500	1:5000	1:10,000	1:20,000	1:40,000	1:80,000
IgG1	2.012	1.322	0.722	0.355	0.175	0.092	0.047
IgG2a	0.765	0.398	0.212	0.099	0.047	0.022	0.010
	Amphotericin B (AmpB) group						
Isotype	1:1000	1:2500	1:5000	1:10,000	1:20,000	1:40,000	1:80,000
IgG1	1.896	1.034	0.492	0.238	0.136	0.075	0.038
IgG2a	0.766	0.398	0.215	0.109	0.052	0.024	0.014
	WTP/AmpB group						
Isotype	1:1000	1:2500	1:5000	1:10,000	1:20,000	1:40,000	1:80,000
IgG1	1.744	0.903	0.454	0.226	0.126	0.072	0.035
IgG2a	0.776	0.387	0.223	0.111	0.048	0.026	0.014
	A4 group						
Isotype	1:1000	1:2500	1:5000	1:10,000	1:20,000	1:40,000	1:80,000
IgG1	1.022	0.577	0.298	0.164	0.087	0.044	0.019
IgG2a	2.033	1.166	0.612	0.303	0.166	0.094	0.052
	A4/AmpB group						
Isotype	1:1000	1:2500	1:5000	1:10,000	1:20,000	1:40,000	1:80,000
IgG1	0.924	0.455	0.232	0.111	0.056	0.030	0.013
IgG2a	2.015	1.133	0.622	0.325	0.155	0.082	0.050
	A8 group						
Isotype	1:1000	1:2500	1:5000	1:10,000	1:20,000	1:40,000	1:80,000
IgG1	0.944	0.501	0.243	0.125	0.067	0.030	0.016
IgG2a	2.233	1.355	0.706	0.358	0.193	0.095	0.052
	A8/AmpB group						
Isotype	1:1000	1:2500	1:5000	1:10,000	1:20,000	1:40,000	1:80,000
IgG1	0.733	0.378	0.201	0.096	0.055	0.031	0.020
IgG2a	2.322	1.454	0.796	0.390	0.192	0.093	0.054

**Table 2:** Titration curves performed for anti-SLA IgG1 and IgG2a peroxidase-conjugated antibodies using serum samples collected 30 days after the immunotherapeutics. Sera samples were collected from animals (*n* = 8 per group), 30 days after the immunotherapeutics procedures, and used in ELISA to evaluate the anti-parasite humoral reactivity. Microtiter plates were coated with *L*. *amazonensis* SLA (1.0 µg per well), reacted against pooled sera (1:100 diluted in PBS-T) and against IgG1 or IgG2a peroxidase-conjugated antibodies, which were 1:1000 to 1:80,000 diluted in PBS-T. Color-imetrical reactions were developed, stopped and read in a spectrophotometer at 492 nm. The mean of optical density (OD) value for each antibody dilution and mouse group is shown.

	Saline Group						
Isotype	1:1000	1:2500	1:5000	1:10,000	1:20,000	1:40,000	1:80,000
IgG1	2.677	1.897	1.044	0.558	0.277	0.144	0.082
IgG2a	0.709	0.395	0.201	0.094	0.053	0.023	0.017
	Wild-type phage (WTP) group						
Isotype	1:1000	1:2500	1:5000	1:10,000	1:20,000	1:40,000	1:80,000
IgG1	2.455	1.504	0.833	0.423	0.233	0.143	0.083
IgG2a	0.702	0.387	0.221	0.117	0.056	0.031	0.014
	Amphotericin B (AmpB) group						
Isotype	1:1000	1:2500	1:5000	1:10,000	1:20,000	1:40,000	1:80,000
IgG1	2.103	1.322	0.711	0.355	0.177	0.088	0.051
IgG2a	1.335	0.784	0.422	0.211	0.122	0.071	0.033
	WTP/AmpB group						
Isotype	1:1000	1:2500	1:5000	1:10,000	1:20,000	1:40,000	1:80,000
IgG1	1.877	0.993	0.576	0.281	0.153	0.088	0.049
IgG2a	1.277	0.698	0.388	0.202	0.093	0.047	0.024
	A4 group						
Isotype	1:1000	1:2500	1:5000	1:10,000	1:20,000	1:40,000	1:80,000
IgG1	1.165	0.677	0.311	0.158	0.084	0.047	0.023
IgG2a	2.233	1.324	0.733	0.347	0.176	0.094	0.050
	A4/AmpB group						
Isotype	1:1000	1:2500	1:5000	1:10,000	1:20,000	1:40,000	1:80,000
IgG1	0.804	0.396	0.221	0.104	0.058	0.031	0.017
IgG2a	2.033	1.299	0.722	0.363	0.192	0.102	0.048
	A8 group						
Isotype	1:1000	1:2500	1:5000	1:10,000	1:20,000	1:40,000	1:80,000
IgG1	0.789	0.403	0.221	0.109	0.059	0.031	0.022
IgG2a	2.677	1.533	0.811	0.397	0.198	0.096	0.047
	A8/AmpB group						
Isotype	1:1000	1:2500	1:5000	1:10,000	1:20,000	1:40,000	1:80,000
IgG1	0.504	0.289	0.157	0.088	0.051	0.032	0.015
IgG2a	2.778	1.687	0.903	0.408	0.221	0.117	0.066

**Table 3:** In vivo toxicity. The toxicity was evaluated using animals’ sera, when levels of urea, creatinine, alanine aminotransferase and aspartate aminotransferase enzymes were measured, one and thirty days after the immunotherapeutics. Sera of non-infected and non-treated mice (naive) were used as control. Results are shown as mean, standard deviation and by statistical indication evaluated by paired the Student’s *t* test. Abbreviations: wild-type phage (WTP), amphotericin B (AmpB).

Creatinine					
	01 Day		30 Days		
Groups	Mean	Std. Deviation	Mean	Std. Deviation	Paired *t* test
Naive	0.68	0.096	0.78	0.096	0.252
Saline	2.53	0.150	2.90	0.183	0.001
WTP	1.95	0.129	2.08	0.171	0.194
AmpB	2.48	0.330	2.65	0.129	0.457
WTP/AmpB	2.23	0.206	2.40	0.163	0.367
A4	1.35	0.124	1.30	0.082	0.638
A4/AmpB	1.65	0.238	1.63	0.171	0.903
A8	0.95	0.208	1.08	0.250	0.555
A8/AmpB	1.28	0.171	1.40	0.189	0.504
**Urea**					
	**01 Day**		**30 Days**		
**Groups**	**Mean**	**Std. Deviation**	**Mean**	**Std. Deviation**	**Paired *t* test**
Naive	15.00	0.816	16.50	1.291	0.245
Saline	28.25	1.708	30.75	1.689	0.194
WTP	22.50	1.291	24.00	1.633	0.297
AmpB	27.50	1.286	29.75	2.630	0.299
WTP/AmpB	25.75	1.698	26.25	1.711	0.752
A4	20.75	2.217	19.00	1.826	0.293
A4/AmpB	21.25	1.708	21.50	1.291	0.861
A8	16.25	1.258	18.00	2.160	0.367
A8/AmpB	18.25	1.712	18.75	1.708	0.783
**Alanine Transaminase**					
	**01 Day**		**30 Days**		
**Groups**	**Mean**	**Std. Deviation**	**Mean**	**Std. Deviation**	**Paired *t* test**
Naive	10.50	1.291	11.50	1.291	0.182
Saline	24.75	1.708	26.00	1.414	0.239
WTP	19.50	1.288	20.50	1.291	0.495
AmpB	23.75	1.708	24.00	2.160	0.873
WTP/AmpB	21.50	1.279	22.50	1.288	0.391
A4	15.00	1.826	15.00	1.414	1.000
A4/AmpB	17.25	1.708	17.75	1.708	0.761
A8	12.50	1.299	13.00	2.155	0.769
A8/AmpB	14.50	1.274	14.25	2.217	0.873
**Aspartate Transaminase**					
	**01 Day**		**30 Days**		
**Groups**	**Mean**	**Std. Deviation**	**Mean**	**Std. Deviation**	**Paired *t* test**
Naive	12.25	1.708	13.50	1.289	0.239
Saline	23.50	1.271	25.50	1.291	0.219
WTP	18.50	1.280	20.75	1.708	0.117
AmpB	22.00	1.826	24.00	0.816	0.182
WTP/AmpB	20.50	1.299	22.25	2.217	0.354
A4	15.75	1.708	15.75	1.714	1.000
A4/AmpB	16.75	1.258	17.75	1.698	0.252
A8	13.50	1.287	14.00	0.816	0.638
A8/AmpB	14.50	1.291	16.25	1.717	0.133

## Data Availability

Data supporting this study are described in the text.
